# Protein Biomarkers for Early Detection of Pancreatic Ductal Adenocarcinoma: Progress and Challenges

**DOI:** 10.3390/cancers10030067

**Published:** 2018-03-07

**Authors:** Alex Root, Peter Allen, Paul Tempst, Kenneth Yu

**Affiliations:** 1 Molecular Biology Program, Memorial Sloan Kettering Cancer Center, New York, NY 10065, USA; roota@mskcc.org; 2 Department of Surgery, Memorial Sloan Kettering Cancer Center, New York, NY 10065, USA; allenp@mskcc.org; 3 Department of Medicine, Weill Cornell Medical College, New York, NY 10065, USA; 4 Department of Medicine, Memorial Sloan Kettering Cancer Center, New York, NY 10065, USA

**Keywords:** pancreatic ductal adenocarcinoma, early detection, biomarkers, blood test, ELISA, mass spectrometry, circulating DNA, thrombospondin, *CA19-9*, KRAS

## Abstract

Approximately 75% of patients with pancreatic ductal adenocarcinoma are diagnosed with advanced cancer, which cannot be safely resected. The most commonly used biomarker *CA19-9* has inadequate sensitivity and specificity for early detection, which we define as Stage I/II cancers. Therefore, progress in next-generation biomarkers is greatly needed. Recent reports have validated a number of biomarkers, including combination assays of proteins and DNA mutations; however, the history of translating promising biomarkers to clinical utility suggests that several major hurdles require careful consideration by the medical community. The first set of challenges involves nominating and verifying biomarkers. Candidate biomarkers need to discriminate disease from benign controls with high sensitivity and specificity for an intended use, which we describe as a two-tiered strategy of identifying and screening high-risk patients. Community-wide efforts to share samples, data, and analysis methods have been beneficial and progress meeting this challenge has been achieved. The second set of challenges is assay optimization and validating biomarkers. After initial candidate validation, assays need to be refined into accurate, cost-effective, highly reproducible, and multiplexed targeted panels and then validated in large cohorts. To move the most promising candidates forward, ideally, biomarker panels, head-to-head comparisons, meta-analysis, and assessment in independent data sets might mitigate risk of failure. Much more investment is needed to overcome these challenges. The third challenge is achieving clinical translation. To moonshot an early detection test to the clinic requires a large clinical trial and organizational, regulatory, and entrepreneurial know-how. Additional factors, such as imaging technologies, will likely need to improve concomitant with molecular biomarker development. The magnitude of the clinical translational challenge is uncertain, but interdisciplinary cooperation within the PDAC community is poised to confront it.

## 1. Unmet Needs for Early Detection of Pancreatic Ductal Adenocarcinoma

Pancreatic ductal adenocarcinoma (PDAC) is an aggressive, chemorefractory and recalcitrant cancer and the number of deaths it causes has recently surpassed breast cancer [1]. PDAC is often detected when it is in a relatively advanced stage with only approximately 1 in 4 patients having resectable tumors [2]. Early detection when PDAC is at Stage I or II can provide a window of opportunity when the disease can be stopped [3]. The experience in Japan with pancreatic cancer registry shows that early detection of PDAC improves overall survival [4]. Therefore, development of an early diagnostic test is a major unmet need. A promising source for an early diagnostic test is blood-based molecular biomarkers, which include proteins, nucleic acids, autoantibodies, aberrantly glycosylated antigens, exosomes, circulating tumor cells, and metabolites or panels thereof. These ideal, non-invasive biomarkers would also be universally present in advanced pre-cancerous lesions (PanIN2-3, IPMN with high-grade dysplasia, carcinoma in situ), specific, sensitive, inexpensive, rapid, and practical to perform [5]. This article aims to describe recent progress and remaining challenges for this research-in-translation and for protein biomarkers more generally.

## 2. Development of Molecular Biomarkers

**Figure 1:**
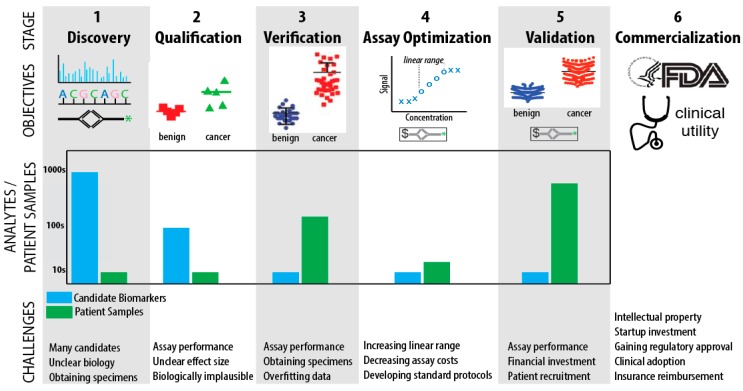
Illustration of the biomarker pipeline. Discovery of biomarkers typically utilizes mass spectrometry, nuclear acid sequence analysis, or ELISAs. In Qualification and Verification the objective is to find biomarkers or a panel of biomarkers that can separate cancer from benign disease and normal controls. During Assay Optimization the linear range of the assay is rigorously assessed and improved, if possible. There must be a representative diversity of patient samples chosen for Assay Optimization, so that the full spectrum of sample heterogeneity that may be ultimately encountered in the clinic is properly optimized for. For Validation, a prospective clinical trial is conducted and cost effectiveness is further assessed. Commercialization may proceed in concert with Validation and requires a demonstration of clinical utility. As the pipeline progresses, there is a sharp decrease in the number of biomarker candidates analyzed and a corresponding increase in the number of patient samples analyzed.

Translating promising research on biomarkers to clinically useful tests is a complex interdisciplinary problem with few successes [6]. In an insightful perspective article Rifai and colleagues describe the pipeline for development of molecular biomarkers consisting of six major steps: discovery, qualification, verification, research assay optimization, clinical validation, and regulatory approval & commercialization [7]. In our opinion, it remains the best roadmap to guide this multiyear process and we have illustrated it in Figure 1, including challenges and pitfalls. In discovery (step 1), the objective is to profile model systems or patient samples to nominate candidates with differential abundance between PDAC and normal, PDAC and chronic pancreatitis, or PDAC and other non-adenocarcinoma neoplasms of the pancreas. The vast majority of published biomarker research ends at this stage and, given the thousands of studies already published, is arguably useless unless it proceeds to subsequent stages [7]. Qualification (step 2) tests the detectability and discriminatory power of candidate biomarkers in human samples, usually bodily fluids, e.g., serum or plasma, and compares cases to controls using targeted technologies such as PCR or LC-MS/MS with multiple reaction monitoring (MRM) or parallel reaction monitoring (PRM). Verification (step 3) involves assessing the sensitivity and specificity of the candidates in large numbers of samples, typically 100 or more. Research assay optimization (step 4) for nucleic acid, protein, and metabolite candidate biomarkers requires development and characterization for immuno- or mass spectrometry-based assays. Research assay optimization will likely include a second-step where individual assays are optimized together as a panel [8]. Clinical validation (step 5) requires design of a prospective trial with regulatory approval in mind. Pepe and colleagues provided a rigorous system to guide study design with their PRoBE guidelines [9]. Regulatory approval may occur in concert with commercialization (step 6). 

The search for biomarkers is motivated by the knowledge that early detection of PDAC will be clinically useful because timely surgical resection can lead to a cure [4]. Cohen and colleagues note that a clinically useful screening test is likely to uncover a greater proportion of earlier-stage tumors because the individuals are asymptomatic and have smaller tumors, which are easier to cure than the typical patients presenting with PDAC today [10]. Additionally, from a theoretical evolutionary dynamics perspective, the tumor population size is a critical determinant of the probability it will evolve resistance in response to therapy and, therefore, early detection of consequently smaller tumors has tremendous implications for eradication by neo-adjuvant therapy and surgery [11].

There is vigorous debate in the community about how feasible early detection of PDAC is for different risk groups. Pannala and colleagues argued “since the incidence of pancreatic cancer is low, screening for asymptomatic cancer in the general population will not be feasible”, and instead argue for a two-tiered screening strategy of stratifying patients into high-risk cohorts based on family history, recent-onset diabetes, or other factors to be determined [12]. We note that in some cases screening for rare diseases is feasible and clinically useful. Phenylketonuria has low incidence, but screening for it is a major medical accomplishment [13], so rarity alone is not dispositive. Panalla and colleagues describe concrete numbers:

“The age-adjusted incidence of pancreatic cancer in subjects ≥50 years of age is 38/100,000. If a test with 99% sensitivity and 99% specificity for pancreatic cancer is used to screen 100,000 subjects ≥50 years of age, the test would identify nearly all pancreatic cancers in the population screened (*n* = 37). However, the test would also falsely identify nearly a 1000 subjects as having pancreatic cancer.”

[12]

Pannala and colleagues are correct to point out that even a diagnostic test with excellent specificity and sensitivity seems to have an impossible, high bar. However, we would add that evaluation of the utility of a biomarker is complex and requires cost-effectiveness analysis that includes a holistic evaluation, including what happens subsequently to those individuals who take the test. Of particular importance for rare diseases is the cost of false-positives. If the test itself is inexpensive, and the consequence of a false positive is a non-invasive imaging test, it is a less costly outcome then if the patient receives an unnecessary invasive biopsy. Therefore, it is an oversimplification to merely require that a biomarker achieves cutoffs for specificity and sensitivity. Careful cost-effectiveness analysis of the test itself and its consequences and alternatives is needed to assess clinical utility. The frameworks of decision analysis and economic evaluation were developed to better evaluate clinical utility than accuracy measures, such as sensitivity and specificity [14]. Andrew Vickers provided a refinement to simplify decision analysis with decision curve analysis [15], noting that traditional accuracy measures such as sensitivity, specificity and positive predictive value are useful in the early stages of biomarker development [15]. Ghatnekar and colleagues provided a detailed examination of these advanced cost-effectiveness concepts, using quality-adjusted life years (QALY), incremental cost per QALY gained (ICER), and cost-effectiveness acceptability curve (CEAC) [16]. They modeled these concepts applied to PDAC biomarker panels, concluding that for high-risk patients a serum biomarker signature was highly desirable [16].

The most probable path for achieving clinical usefulness in the near-term is to follow the two-tiered screening strategy of Pannala and colleagues, whereby firstly, high-risk patients are identified and then screened [12]. High-risk patients comprise 10–15% of PDAC and include those with pancreatitis, intraductal papillary mucinous neoplasms, recent onset diabetes, *CDKN2A*, *BRCA1/2*, *PALB2*, *STK11*, *ATM*, *CTFR* germline mutations, and family history with two or more first-degree relatives having PDAC [17,18]. Given that screening high-risk patients is the intended use, it is an important question as to whether these patients behave as a distinct subset with different biomarker panel performance than non-high-risk cases. Norris and colleagues compared the genomic progression of familial vs. sporadic PDAC and concluded that it is the same [19]. Therefore, both sporadic and familial cases can be used to develop biomarkers, even though the intended use is screening high-risk cases. This is significant because high-risk cases are rarer and limiting patient accrual to this subset would make it much more difficult to reach the hundreds of cases needed for statistical power. The test itself is likely to be a panel of biomarkers [8]. An important question for the field is: how high should numerical cutoffs for area-under-curve, sensitivity, and specificity be for high-risk cohorts? 

We recommend that efforts to find biomarker panels for non-high-risk patients continue even though they do not represent the population for intended use because: (1) the genomic progression of PDAC appears to be the same whether patients have familial or sporadic PDAC; therefore, studying both groups together is pragmatic for patient recruitment; (2) the definition of “high-risk” continues to change as more is learned about gene-disease and other risk factors; (3) candidate biomarker levels could be useful to stratify patients into high risk groups for surveillance, and therefore, the search for early detection biomarkers in non-high-risk cases could help predict risk; (4) improvements in sequencing and mass spectrometry technologies may uncover truly PDAC-specific signatures with >99% sensitivity and specificity; such revolutionary developments have happened before, as occurred with newborn screening for in-born errors of metabolism; (5) the enormous potential cost savings of an early detection test for PDAC more than compensates for the apparently low probability that a general population screening test can be developed—in other words, the risk is worth the reward. 

## 3. Recent Advances in Protein Biomarkers for Early Detection of PDAC

The research community has devoted much time and resources to developing biomarkers for early detection of PDAC with better performance than *CA19-9* [3]. Current clinical practice uses an ELISA for *CA19-9*, which is a carbohydrate found on multiple carrier proteins, whose identities may themselves have diagnostic significance [20]. However, *CA19-9* is not detectable in 5–10% of patients who lack fucosyltransferase activity due to germline variants [21]. *CA19-9* is useful for monitoring response to therapy, but not very useful as an early detection biomarker [20]. Whether it should be part of a biomarker is uncertain. However, it is routinely measured as part of clinical care of research cohorts, and therefore, should be included in biomarker studies. 

Biomarkers for diagnosis, treatment prediction, and prognosis of PDAC were reviewed by Chari and colleagues [5], Bunger and colleagues [22], Loosen and colleagues [23], Gallego and colleagues [24], and exosome biomarkers by Li and colleagues [25]. Four key studies nominating primarily protein diagnostic biomarkers of particular—albeit highly subjective—interest to us, which report outperforming *CA19-9* alone, are briefly summarized in Box 1, and their performance characteristics are shown in Table 1. We note at the outset that most are multivariate panels including *CA19-9*.

Box 1 **Recently reported proteomic biomarker panels with better performance than *CA19-9.***

**Cohen 2017** [10]: Found that combining ctDNA testing for KRAS mutations in combination with 4 plasma proteins (*CA19-9*, *CEA*, *HGF*, *OPN*) outperformed *CA19-9* alone in discriminating PDAC from normal controls, chronic pancreatitis, and other benign pancreatic diseases. A disappointing 64% sensitivity begs the question as to whether including additional biomarkers or optimizing the assays may improve performance. Evaluation in an independent test set is desirable. **Capello 2017** [21]: Began with 17 protein biomarker candidates from previous studies and validated several that can distinguish PDAC from controls and chronic pancreatitis (*TIMP1*, *LRG1*, *REG3A*, *IGFBP2*, *COL18A1*, *TNFRSF1A*), finding *TIMP1 + LRG1 + CA19-9* outperformed *CA19-9* alone. Evaluation in an independent test set is desirable.**Kaur 2017** [26]: Performed an in-depth analysis of the literature and observed that *MUC5AC* has favorable biomarker properties: secretion, high over-expression in PanIN, and numerous epitopes. They developed an in-house ELISA against *MUC5AC*, finding that *MUC5AC + CA19-9* outperforms *CA19-9* alone in two, large independent validation cohorts.**Kim 2017** [27]: Began with a state-of-the-art cell reprogramming model of PDAC and sophisticated systems biology network analysis to nominate thrombo-spondin-2 (*THBS2*); when combined with *CA19-9* it outperformed *CA19-9* in discriminating PDAC from controls and chronic pancreatitis. The two biomarker panel was evaluated in a discovery phase and two validation phases, however, the number of Stage I/II patients was 0 in the discovery phase, and 7 Stage I and 34 Stage II in validation set A.

Molecular profiling of PDAC model systems is a widely used strategy for biomarker discovery. Cell lines, mouse models, and organoids can satisfactorily recapitulate disease progression and be more amenable to intervention and analyses in the laboratory than precious samples from human patients. In 2008 Faca and colleagues took an innovative approach by using *Pdx1-Cre Ink4a/Arf^lox/lox^* and *Kras^G12D^ Ink4a/Arf^lox/lox^* genetically engineered mice in order to sample blood from normal, early PanIN, advanced PanINs, locally advanced cancer, and metastatic cancer [30]. Using a tour-de-force proteomics approach to identify biomarkers directly in plasma they followed a major protein immune-depletion strategy, isotopic labeling, and extensive pre-fractionation before identifying 1442 unique proteins with LC-MS/MS. Additional candidates were included with mRNA analysis; absence in the mouse liver proteome; high expression ratio PDAC/PanIN; not annotated for acute-phase reactants or coagulation; and having a human orthologue—resulting in 45 proteins—a handful of which had commercially available antibodies or ELISA kits. These were quantified in mouse pancreas tissue or blood and researched for previous analysis in published studies. Subsequently 10 proteins were tested in 30 human PDAC sera, 20 matched controls, and 10 chronic pancreatitis cases. Performance of the markers distinguishing PDAC from controls and chronic pancreatitis revealed statistically significant biomarker candidates *ALCAM1*, *TIMP1*, *ICAM1*, *REG3*, *IGFBP4* panel plus *CA19-9*. The authors also note a striking concordance between mouse and human markers, with 8 out of 9 concurring. 

Given the plethora of diagnostic biomarker discovery studies already published it can be productive and tremendously cost-effective to analyze the literature and combine reported candidate markers in a panel, instead of performing a costly discovery step 1, and thereby begin at step 2 (qualification). Poruk and colleagues employed this strategy and assessed the utility of secreted proteins *OPN* (osteopontin) and *TIMP1* (tissue inhibitor of metalloproteinase 1) plus *CA19-9* as diagnostic or prognostic biomarkers using commercially available ELISA kits [28]. Serum from 220 subjects was analyzed and an iterative classification tree analysis determined cut-points of *CA19-9* above 37U/mL followed by *OPN* 25 ng/mL and *CA19-9* below 37U/mL further split by *TIMP-1* level of 411 ng/mL. Panel performance plus *CA19-9* is summarized in Table 1. Testing performance of these markers in additional validation datasets is highly desirable.

Comprehensive genomic analyses of PDAC tissue and cell lines is leading to a new understanding of disease biology [31] and overexpressed genes are a sensible source for biomarker candidates. Kaur and colleagues began with this insight and analyzed over-expression data for the most highly expressed mucin gene in PDAC relative to benign controls, nominating *MUC5AC*. They also observed that it has additional desirable biomarker attributes including high expression in early disease stage (PanIN), secreted protein, and containing multiple epitopes [26]. They developed a highly sensitive sandwich ELISA (University of Nebraska Medical Center) using a monoclonal antibody provided by Jacques Bara (INSERM). Diagnostic training set samples were obtained from University of Pennsylvania Medical Center; validation set 1 was obtained from The Mayo Clinic; validation set 2 from University of Pennsylvania Medical Center; and tissue microarrays from Anirban Maitra (Johns Hopkins). This study is to be commended for truly community-wide collaborative efforts. Diagnostic performance of *MUC5AC*, *CA19-9* and combination panel is shown in Table 1.

In a complementary approach to developing protein biomarkers in blood (serum or plasma), urine has also been used as source for biomarkers. Radon and colleagues analyzed 18 PDAC, chronic pancreatitis, and normal controls by mass spectrometry [29]. Data analysis revealed LYVE1, REG1A/B, and TFF1 having discriminatory power in both genders. Presence of full-length proteins was confirmed by western blot in urine. ELISA assays for 488 urine samples were done with performance characteristics shown in Table 1. Data was randomly partitioned into training and test sets (70%/30%). Cutpoint optimization was performed on the training set using the R pROC software package, adjusted by creatine levels and age into a 5-part panel logistic regression model. Testing in PDAC tissues using IHC revealed the presence of REG1A, TFF1, with LYVE1 positive staining in lymph nodes. Testing these urine biomarkers in combination with blood-based biomarkers or miRNA urine biomarkers [32] is highly desirable. 

In-depth analysis of the literature and datasets is required to put new information in context; performing this scholarly work, termed “context placement”, can help make clinical research more useful [33]. Capello and colleagues revisited the data in Faca 2008 [30] and performed an analysis of the literature to arrive at 17 candidates plus *CA19-9* using commercially available ELISA kits for 16 proteins and developing their own ELISA for 1 protein. Seven candidates passed qualification (step 2) and verification (step 3) in multiple, independent datasets that considerably advanced towards completion of research assay optimization (step 4) and clinical validation (step 5) [21]. Performance of the biomarker candidates is shown in Table 1. A particularly noteworthy statistical reporting advance was made in this study, and involves finding and reporting: sensitivity at 95% specificity and specificity at 95% sensitivity, where most studies only report maximum sensitivity and specificity; and evaluation of biomarkers and panels in 3 totally independent validation datasets where most studies only report a single validation set. 

The development of organoids recapitulating PDAC is a major recent achievement and one of their numerous good uses is biomarker development [2]. Of comparable magnitude, the network biology paradigm has advanced analysis of biological systems to consider how biomolecules act multilaterally to achieve complex phenotypes rather than a reductivist view of single molecules acting unilaterally [34]. Putting both of these advances together, Kim and colleagues used the PanIN organoid model “10–22 cell-derived” and cross-referenced their 107 secreted proteins with: low abundance in the healthy human plasma proteome; RNA-seq datasets; commercial availability of ELISAs; and presence in the integrated networks for TFG-beta and integrin signaling, which drive PDAC [27]. This resulted in an investigation of *MMP2*, *MMP10* and *THBS2* (thrombospondin 2) plus *CA19-9*. Initial assessment revealed the discriminatory power of *THBS2,* and this was subsequently validated in two phases with performance characteristics of the two-panel marker shown in Table 1. This study is also notable for its statistical designs: adherence to PRoBE guidelines; using 3 different lot numbers for ELISA kits; blinding experimenters with de-identified samples; performing ELISAs in multiple laboratories; and comparison of PDAC vs. multiple benign diseases. While a large number of samples were tested in validation sets A and B, most samples were Stage III/IV, so further testing of this panel in Stage I/II cohorts is desirable in order to establish performance in the intended use of early detection of Stage I/II PDAC.

Mutations in *KRAS* are highly recurrent in PDAC and drive tumorigenesis from the earliest stages, making them a highly desirable target biomarker for early detection [35]. Cohen and colleagues combined measurement of *KRAS* mutations in ctDNA with several protein biomarkers *CEA*, *HGF*, *midkine*, *OPN*, and *prolactin* plus *CA19-9*. Their best performing combination assay consisted of *ctDNA*, *CA19-9*, *CEA*, *HGF*, and *OPN* with performance characteristics summarized in Table 1. They also found that the panel had prognostic value beyond clinical and histopathology in current use—patients with poorer survival were more likely to have a positive test. Overall, this study attempted to demonstrate that combining nucleic acid and protein candidate biomarkers is a promising approach; however, further research is needed to establish this. 

There are a number of take-aways from the studies highlighted in Box 1. Commonalities include sophisticated use of model systems or existing literature for candidate discovery that advance the field beyond the use of traditional cell lines or the difficulties of direct discovery in plasma. Most studies used plasma or serum rather than bodily fluids such as urine, pancreatic juice, bile, or ascites. The validation method of choice is almost always ELISAs and frequently the commercial availability of ELISA kits is a make-it-or-break-it filter for advancing a candidate through the biomarker pipeline. Diversity in approaches among studies included their measurement technologies, the number and size of validation datasets, the specific reporting of statistics, and the case-control comparisons. All studies found improved performance with a multivariate panel including *CA19-9* over any one marker alone. Most studies assume that *CA19-9* is “an anchor marker”, and therefore will increase performance with candidate markers and in combination [21,36]; however, this assumption should be questioned, and we recommend authors compute performance for all combinations of all biomarker candidates. Despite the promising performance of these studies shown in Table 1, significant challenges remain. 

## 4. Biomarker Challenges

### 4.1. Challenges in Discovery, Qualification, and Verification

There have been numerous papers published with PDAC candidate biomarkers, even though this research is difficult and time-consuming [23,24], because the human plasma proteome contains proteins spanning seven orders of magnitude in concentration, the heterogeneity of normal individuals, and disease heterogeneity that all present enormous analytical challenges [37]. Arguably, the field is also overwhelmed with published candidates. Harsha and colleagues examined more than 5000 published articles on PDAC and performed meta-analysis to filter towards a list of 60 proteins that antibodies were developed against and raised the question of whether any more biomarker discovery studies should be performed in the field [38]. In our opinion biomarker discovery studies should continue provided they proceed through the first 3 steps of the pipeline (discovery, qualification, verification) with adequate numbers of samples, because technological and methodological advances have increased assay sensitivity such that low abundant biomarkers that were invisible in past discovery scans may now be discovered. Similarly, new model systems including organoids and genetically engineered mice should continue to be explored. 

An issue for discovery studies is that the methods and reporting of statistics is not uniform, which makes comparison difficult and necessitating meta-analyses and head-to-head comparisons. Studies should also report performance by stage (IA, IB, II, III, IV), because the intended use is detection of stages I/II. Guidelines for studies have been proposed [9]; however, implementing them in practice is challenging [39]. Perhaps less widely known but equally significant are the statistical constraints on the development of multivariate classifiers from large numbers of variables and relatively small sample sizes that lead to tradeoffs in the stability of the variables chosen and classification error, termed “the curse of dimensionality” [40]. Best practices in biomarker statistics require researchers to avoid over-fitting, cherry-picking, and other analysis pitfalls [9]. There has been enormous progress in methodology, and biomarker panels appear to be the best way forward [8].

### 4.2. Challenges in Assay Optimization, Validation, and Commercialization

Given that there are now multiple reported early detection panels for PDAC with area-under-curve, sensitivity and specificity above 0.75, and commercially available ELISAs, what challenges must be overcome to advance along the biomarker pipeline? We believe top priorities for the field are: (1) optimize assays and assay panels for the top candidates; (2) assess the top candidates as panels in independent cohort sets; (3) propose threshold values for accuracy metrics (AUC, sensitivity, specificity, PPV, NPV) for the intended-use of screening high-risk groups. What can we learn from past experience with biomarker failures? 

In a blistering critique of the biomarker field, Scott Kern argues for a wholesale change in practices and a recognition of why biomarkers fail including “lack of statistical significance, hidden structure in the source data, a technically inadequate assay, inappropriate statistical methods, unmanageable domination of the data by normal variation, implausibility, deficiencies in the studied population or investigator system, and its disproof or abandonment for cause by others” [41]. Ioannidis categorized biomarker failures into 4 categories: clinical reversal (type A), validation failure (type B), non-optimized clinical translation (type C), and promotion despite non-promising evidence (type D) [42]. It is important to decide whether top candidates are being abandoned for cause and if so, state this clearly in a publication. Is there a validation failure (type B)? Otherwise, without an attempt at completing the biomarker pipeline the markers will fail due to non-optimized clinical translation (type C failure.).

Past experience has taught us that the vast majority of biomarker research does not advance past step 3 verification, despite reportedly excellent performance of biomarker candidates [6,7]. How do we improve this bottleneck? A priority for funding should be to incentivize assay optimization and facilitate sample repositories for scaling up. We recommend that leadership in the United States from the Early Detection Research Network (NCI) continue to help facilitate this, in particular by providing funding for assay optimization and validation. Even though commercially available assays exist, their performance characteristics may be inadequate for approval under FDA requirements. Assays may have high batch-to-batch variation, poor signal-to-noise, inadequate limit-of-detection, or a narrow linear range. 

Testing panels of top candidates in independent validation datasets is the best way see whether they really work [41]. Difficulties in obtaining patient samples for analysis have been ameliorated by the creation of the Mayo Clinic Prospective Resource for Biomarker Validation and Early Detection of Pancreatic Cancer, which promises only a page of paperwork [43]. Another source for patient cohorts is surveillance studies of high-risk populations, where surveillance is typically imaging-based [18,44,45]. Consortia such as the National Familial Pancreatic Tumor Registry also seek to identify and study these patients. Large surveillance studies have been conducted on high-risk cohorts with demonstrated survival benefits [18]. Potjer and colleagues examined performance of a serum-based proteomic panel for early detection in a high-risk cohort undergoing imaging-based surveillance for PDAC [46]. This study design is one way forward because it incorporates intended use of screening high-risk patients, as well as imaging. The difficulty is that high-risk cohorts are smaller than all PDAC cases. As was stated earlier, sporadic and familial pancreatic cancers share common genomic driver events [19]. Scaling up may require creation of another prospective resource for biomarker validation in addition to Mayo Clinic’s.

There are also socio-economic challenges to translate biomarkers. For example, given that there are already commercially developed ELISA kits for a number of promising candidate markers and these markers typically perform best as part of a panel, it is natural to ask whether the regulatory environment is adequately incentivizing development of a marker panel consisting of diagnostic assays from multiple companies. The government may very well need to incentivize collaboration among companies to optimize and validate assay panels. Experience in promoting collaboration among academia, foundations, and industry in developing novel therapies was recently reviewed by Ramsey and colleagues and contains useful lessons that may be applied to translating biomarker research [47]. Stern and colleagues discuss incentives for biomarker development, recommending that NIH target and increase funding [48]. They also mention the FDA’s Accelerated Approval Program [48]. To better incentivize biomarker development, collaboration among industry, and recognize the high risks involved in this endeavor, we boldly recommend an additional five years of patent protection for early detection tests. 

## 5. Outlook

Progress has been achieved discovering and assessing biomarkers for early detection of PDAC. The field has nominated several panels with sensitivity and specificity above 0.75, with most panels not tested in independent validation sets, and most combinations of candidate markers not yet assessed. In our opinion, investment in assay optimization and independent validation studies should be a moonshot priority. There are significant challenges remaining including: optimizing assay and panel performance; scaling up validation studies to include hundreds of cases and controls; variability and hidden data structure that result in poor generalizability of panel performance in independent cohorts; and furthering collaboration among interdisciplinary teams from research, industry, and government. It is also important for the field to learn from previous experience, so as not to repeat mistakes [41]. Surveillance studies of high-risk individuals that use imaging can incorporate blood sampling for biomarker development, but this adds complexity and the necessity for teams with multiple areas of expertise. Case-control validation cohorts incorporating all PDAC risk groups with an intended use of a two-tiered strategy of identifying and screening high-risk patients is the most probable path forward. The initial application of biomarkers is likely to be in the high-risk population. It has nothing to do with biology, but everything to do with statistics. For a given sensitivity and specificity, the performance of a biomarker is going to vary greatly based on the disease incidence in the population screened. Once a biomarker panel is developed, it will first be used to monitor high-risk patients, followed by confirmatory tests (imaging, endoscopy, etc.). If the test is really good, then it could be used more broadly in populations where the incidence is lower (for example, older patients without other risk factors, etc.). Despite many challenges, the progress made to-date in finding biomarkers for early detection of PDAC provides optimism and invigoration to the field.

## Figures and Tables

**Table 1:** Summarized performance of biomarker candidates. Listed here are published performance metrics for cancer vs. healthy or benign controls and cancer vs. chronic pancreatitis in independent validation datasets when available, across all stages reported. Studies do not always make these basic statistics available in an easy-to-read table and not all numbers are directly comparable.

Study	*n* (Training; *val1*; *val2*) Training Stage I/II Val. A Stage I/II Val. B Stage I/II Val. C Stage I/II	Biomarkers(s)	Cancer vs. Control *AUC*, *SN*, *SP*	Cancer vs. CP *AUC*, *SN*, *SP*
Poruk 2013 [28]	220; 0; 0 ^ 7/35 Stage I/II na na	*CA19-9*	92, 84, 88 *^,^^	*
		*OPN*	72, na, na *^,^^	*
		*TIMP1*	77, na, na *^,^^	*
		*CA19-9 + OPN + TIMP1*	na, 87, 91 *^,^^	*
Radon [29]∇	134; 58 4/14 Stage I/II *Unclear*	*LYVE1*	84, 68, 92; na, na, na	73, 77, 62; na, na, na
		*REG1A*	75, 75, 69; na, na, na	72, 75, 70; na, na, na
		*TFF1*	70, 79, 53; na, na, na	65, 77, 56; na, na, na
		*panel + creatine + age*	90, 82, 89; 93, 80, 77	83, 86, 67; 85, 100, 50
		*panel + plasma CA19.9*	na, na, na; 97, 88, 96	na, na, na; 87, 75, 94
Kaur 2017 [26]	346; 94; 321 70 Stage I/II *Unclear* *Unclear*	*MUC5AC*	84,70,83; 70,68,73; 74,65,83 ^§^	*
		*CA19-9*	57,48,67 ^§^; na, na, na; na, na, na	*
		*MUC5AC + CA19-9*	86,72,85 ^§^; na, na, na; na, na, na	*
Capello 2017 [21]	121; 30; 142; 35~ *Unclear* 10 Stage I/II 42 Stage I/II 21 Resectable	*CA19-9*	88, 73, 23~	82, 29, 24~
		*TIMP1*	81, 41, 50~	73, 22, 33~
		*LRG1*	85, 43, 25~	68, 11, 12~
		*TIMP1 + LRG1 + CA19-9 (“OR” rule)*	96, 85, 67~	89, 45, 54~
Kim 2017 [27]	20; 189; 537 0 Stage I/II 7/34 Stage I/II 4/37 Stage I/II	*CA19-9*	85, 69, 100; 58, 78, 99	77, na, na; 82, na, na
		*THBS2*	84, 33,96; 58, 94, 75	73, na, na; 73, na, na
		*CA19-9 + THBS2*	96, 74, 96; 76, 88, 93	84, na, na; 87, na, na
Cohen 2017 [10]	na; 403; 0 na 29/102 Stage I/II	*ctDNA*	na, 30, na	na, na, na
		*CA19-9*	na, 49, na	na, na, na
		*CEA + HGF + OPN*	na, 18, na	na, na, na
		*ctDNA + CA19-9*	na, 60, na	na, na, na
		*ctDNA + CEA + HGF + OPN*	na, 42, na	na, na, na
		*CA19-9 + CEA + HGF + OPN*	na, 54, na	na, na, na
		*ctDNA+CA19-9+CE+ HGF +OPN*	na, 64, 99.5	na, na, na

*n* total cases and controls, *val1/2* validation datasets, *SN* sensitivity, *SP* specificity, *CP* chronic pancreatitis, *na* not available). * control and chronic pancreatitis combined, ^ performance on training set, ~3rd independent validation dataset used and performance reported for combined validation datasets. ^§^ Early pancreatic cancer vs. healthy controls. ∇ Stage I/II vs. healthy control or chronic pancreatitis.
